# Estimation of environmental effects and genetic parameters of carcass traits on Chikso (Korean brindle cattle)

**DOI:** 10.5713/ajas.19.0136

**Published:** 2019-08-03

**Authors:** Byoungho Park, Tae Jeong Choi, Mi Na Park, Sang-Hyon Oh

**Affiliations:** 1 National Institute of Animal Science, RDA, Cheonan 31000, Korea; 2 Department of Agriculture, Food, and Resource Sciences, University of Maryland Eastern Shore, Princess Anne, MD 21853, USA

**Keywords:** Heritability, Genetic Correlation, Carcass Traits, Chikso, Korean Brindle Cattle

## Abstract

**Objective:**

The purpose of this study was i) to identify the characteristics of carcass traits in Chikso by gender, region, age at slaughter, and coat color using the carcass data collected from the nationwide pedigree information and coat color investigation, and ii) to estimate genetic parameters for breed improvement.

**Methods:**

A linear model was used to analyze the environmental effects on the carcass traits and to estimate genetic parameters. Analysis of variance was performed using TYPE III sum of squares for the unbalanced data provided by the general linear model procedure. Variance components for genetic parameters was estimated using REMLF90 of the BLUPF90 family programs.

**Results:**

Phenotypic performance of carcass weight (CW), eye muscle area (EMA), and backfat thickness (BF) in Chikso were lower than those of Hanwoo. This is a natural outcome because Hanwoo have undergone significant efforts for improvement at the national level, a phenomenon not observed in Chikso. Another factor influencing the above outcome was the smaller population size of Chikso compared to that of Hanwoo’s. The heritabilities of CW, EMA, BF, and marbling score in Chikso were estimated as 0.50, 0.37, 0.35, and 0.53, respectively, which were was higher than those of Hanwoo.

**Conclusion:**

Based on the genetic parameters that were estimated in this study, it is expected that the carcass traits will improve when the livestock research institutes at each province conduct small-scale performance tests and the semen is provided to farmers after selecting proven bulls using the state-of-art selection technique such as genomic selection.

## INTRODUCTION

In South Korea, Hanwoo (Korean Native Cattle; *Bos taurus coreanae*) has been raised as beef cattle with a yellowish-brown coat. Other cattle such as Chikso (Korean Brindle Cattle), Heugu, and Jeju Black were reported as domestic beef cattle breeds by the Domestic Animal Diversity Information System of the Food and Agriculture Organization [1,2]. Today, the four indigenous Korean breeds include Hanwoo, Chikso, Heugu, and Jeju Black [3]. It is reported that Chikso cattle are brindle with black color on a yellow background; however, Heugu and Jeju Black cattle only show a black coat color [1,4]. Recent molecular biology studies on these breeds have shown that Hanwoo and Chikso are very close genetically [5,6].

Hanwoo has improved overall by way of supplying semen to producers while proven bulls are being selected genetically. However, Chikso improvement has always been implemented as small-scale regional district level improvement projects, never a nationwide project [7,8]. The researches on Chikso mainly focused on genes affecting the coat color [9,10] and how to increase black streaks on the coat [11,12]. Although research on performance improvement in Chikso has not occurred yet, Hanwoo has been studied at the national level to improve beef cuts as well as carcass weight (CW) and intramuscular fat [13].

The National Institute of Animal Science (NIAS) in South Korea, in cooperation with the Korea Animal Improvement Association (KAIA) and the regional district livestock research institutes, is investigating the pedigree information and coat colors of Chikso cattle in the country since 2013. Their projected goal is to establish Chikso as a breed and lay the foundations for industrialization [2]. The Ministry of Agriculture, Food and Rural Affairs is promoting the improvement of the Chikso breed through the rare Korean cattle improvement project.

The purpose of this study was i) to identify the charac teristics of carcass traits in Chikso by gender, region, age at slaughter, and coat color using the carcass data collected from the nationwide pedigree information and coat color investigation, and ii) to estimate genetic parameters for the breed improvement.

## MATERIALS AND METHODS

### Data

The data used in this study was collected by NIAS with the KAIA and the eight regional district livestock-related research institutes that investigated the pedigree information and coat colors of Chikso in the country in 2013. The photographs of the cattle were taken and classified into seven levels according to the standards set by the KAIA; the first level is when the brindle pattern is strongly expressed throughout the body; the second level is when the brindle pattern is expressed in the whole body but the expression level is weak; the third level is when the brindle pattern is expressed partially; the fourth level is no brindle pattern expression with a yellowish brown coat color; the fifth level is no brindle pattern expression with black coat color; the sixth level is no brindle pattern expression with a mixture of yellowish brown and black coat color; the seventh level is black color in eyes, nose, legs, and tail with a yellowish brown coat color [2]. These classifications are for an academic purpose, which don’t have any relation with the authenticity of the Chikso.

When the Chikso were slaughtered, grading results from the Korea Institute for Animal Products Quality Evaluation (KAPE) were derived based on the individual identification numbers. The grading results include the date at slaughter, breed, sex, slaughter house, grading date, CW, eye muscle area (EMA), backfat thickness (BF), marbling score (MS), meat color score, fat color score, texture, maturity, meat yield score, meat yield grade, final grade, etc. For statistical analysis, the individuals born from 2004 to 2016 which received a “pass” grade were used. The individuals that couldn’t be categorized with coat color were removed from the data. In addition, interquartile range criterion was applied for the removal of outliers. Table 1 shows the frequencies of sex, region, birth year, birth season, age at slaughter, and coat color from 2,377 individuals after removing outliers.

### Statistical model

The following linear model was used to analyze the environmental effects on the carcass traits and to estimate the genetic parameters.

(1) $$y_{ijklmno}=\mu +{sex}_{i}+{loc}_{j}+{byear}_{k}+{bseason}_{l}+{sage}_{m}+{cc}_{n}+e_{ijklmno}$$

where *y* is CW, EMA, BF, and MS. *μ* is the overall mean, sex is the sex of Chikso (male, female, or castrate), *loc* is the farm location, *byear* is the year of birth, *bseason* is the season of birth, *sage* is the age at slaughter, *cc* is the coat color, and *e* is the error.

Analysis of variance was performed using TYPE III sum of squares for the unbalanced data provided by the general linear model procedure [14]. Variance components for genetic parameters was estimated using REMLF90 of the BLUPF90 family programs shared by the University of Georgia [15].

## RESULTS AND DISCUSSION

### Culling rate

Among a total of 3,074 Chikso born between 1995 and 2017, 336 individuals (9.07%) culled before slaughter and 2,738 individuals were slaughtered between 2009 and 2018. There were 2,982 (80.51%) individuals with a “pass” grade, 2 (0.08%) with a “fail” grade, and 383 (10.34%) with “undetermined” due to the absence of graders in some of the slaughter house. Table 2 shows the distribution of sex and age of the culled animals before slaughter. The table reported a higher culling rate of females, which is presumed to be because of long occupation periods from producing calves. By age, 99 individuals were culled at 0 year old (less than 12 months), suggesting that the rate of calf death was high. At the age of 0 or 1 years old, which is before the steers and bulls were slaughtered, the culling rate of the cows was higher.

### Overall means by trait

Table 3 shows the means, standard deviations, minimum and maximum values of carcass traits in Chikso. The average CW from 2,377 individuals was 372.6±63.80 kg, the average EMA was 81.97±10.26 cm^2^, the average BF was 11.7±5.3 mm, and the average intramuscular MS was 3.4±2.01.

### Effect of environmental factors

Table 4 shows the analysis of variance for environmental effects on the carcass traits in Chikso. There were significant differences in CW and BF among sex, regions, birth years, and ages at slaughter, but there was no significant difference between birth seasons and coat colors. The EMA showed significant differences among sex, regions, birth seasons, and ages at slaughter but no significant difference between birth years and coat colors. In MS, sex, regions, birth years, birth seasons, and coat colors were significantly different but there was no significant difference for ages at slaughter.

Least square means (LSM) of sex and region effects show ing significant differences in all carcass traits are shown in Table 5 and 6. The LSM of CW in bulls and steers were 404.8 kg and 404.9 kg, which weren’t significantly different.

The 2017 Hanwoo (Korean native cattle) grading results reported by KAPE [14] showed that LSM of CW (439.8 kg) in steers was higher than in bulls (425.6 kg). In general, it would be considered that CW of bulls is higher than of steers when raising them in the same environment, but it seems that there was no significant difference in CW between bulls and steers because they were raised in different environments in different time periods. Likewise, it is presumed that CW in Chikso weren’t significantly different between bulls and steers for the same reason. In particular, this would be one of the reasons why Chikso bulls are used for natural mating, which accounts for the lack of an intensive fattening period.

In EMA, the LSM of bulls, steers and cows was 87.43, 83.94, and 76.14 cm^2^, respectively. These are lower than the EMA means of Hanwoo bulls, steers and cows slaughtered in 2017 reported by KAPE [16], which showed 91.0, 92.1, and 82.9 cm^2^. The LSM of BF in steers and cows were 12.32 mm and 11.92 mm, which were not significantly different, and the LSM of BF in bulls was 5.04 mm. These statistics observe a similar tendency to the average BF in Hanwoo steers and bulls reported by KAPE [16], which were 13.8 mm and 6.2 mm. This shows that the average BF of Chikso was lower than that of Hanwoo. In MS, the LSM of Chikso bulls, steers, and cows were 4.36, 1.37, and 3.40, showing similar tendencies in Hanwoo steers, bulls and cows which had 5.8, 1.4, and 4.3, respectively [16]. These statistics shows that the average MS of Chikso was lower than that of Hanwoo.

Lee and Choy [6] compared the monthly weights of Han woo and Chikso. Hanwoo was heavier at 12 months of age than Chikso, and the difference was larger at 18 and 28 months of age, resulting in their respective carcass traits performance. In the meantime, there has only been a management effort by research institutes at the province level to improve Chikso, not the national level. Therefore, it is feasible that there exists a difference in carcass grades between Chikso and Hanwoo because Hanwoo has experienced consistent and various efforts for improvement at the national level. For the Chikso to undergo carcass trait improvement, it is critical that Chikso also undergoes management efforts at the national level like Hanwoo. As shown in Table 6, the LSM of CW by region were 407.03 kg and 396.94 kg in Jeonnam and Gyeongnam, respectively. The best EMA was 83.98 cm^2^ and 83.70 cm^2^ in Chungnam and Gangwon. The highest BF was 8.38 mm and 8.46 mm in Jeonnam and Gangwon, while the highest MS was 3.73 and 3.34 in Gyeongbuk and Gyeonggi, respectively. Gangwon, Chungbuk, Jeonbuk, and Gyeongbuk (Ulleung) produced their own semen and distributed them; however, the genetic ability of the distributed semen was not confirmed, and it was high proportion of the farmers that produce semen by themselves or do natural service, which seems to show inconsistent performance on regional carcass traits.

The CW, EMA, and BF were not significantly different under the presence of brindle with black color on a yellow background, but MS was significantly influenced. The highest LSM of EMA in the 3rd level of coat color was 3.22 and the highest LSM of EMA in the 4th level was 3.20.

Lee et al [17] compared carcass performance between Chik so and black cattle raised in the inland and found that black cattle were heavier in CW, broader in EMA, thicker in BF and higher in MS. In addition, Choi et al [18] also compared weights at 24 months of age between Chikso and black cattle, and didn’t report any significant difference between them (although the weights of black cattle were heavier numerically).

The livestock-related research institutes at each province are producing semen based on coat color and distributing it to farmers. Therefore, semen with greater carcass traits performance should be provided to them.

### Monthly performance of steers in carcass traits

Carcass traits of steers are directly related to profit in the Chikso industry. Table 7 shows the basic statistics of carcass traits in steers by month when over 100 heads were slaughtered. In comparison with Hanwoo, it was expected that Chikso show longer months of age at slaughter than Hanwoo. However, they were slaughtered the most at 30 months of age, nearly the same age at slaughter of Hanwoo which had about 62% (701 heads) slaughtered between 28 and 32 months of age.

The CWs increased continuously with an increase in months of age at slaughter. The EMA changed little until 31 months of age and slightly increased at 32 months of age.

The BF increased until 31 months of age and decreased at 32 months of age.

In the case of MS, the steady increase was observed based on months of age at slaughter, such as 3.7 at 29 months of age, 4.1 at 30 months of age, 4.3 at 31 months of age, 4.7 at 32 months of age.

### Estimation of genetic parameters

Table 8 shows the estimates of heritability, genetic correlations, and phenotypic correlations of the carcass traits in Chikso. The heritability estimates of the CW, EMA, BF, and MS were 0.50, 0.37, 0.35, and 0.53, respectively. It was higher than the heritability estimate of 0.39 in CW and lower than the heritability estimate of 0.62 in MS reported by Sun et al [19] in Hanwoo. The heritability of CW from this study was estimated higher than that of the study using performance testing records of Hanwoo young bulls (0.37) [20], and using the data from Hanwoo steers [21].

Table 8 shows the genetic and phenotypic correlations be tween traits studied. Sun et al [19] reported that the genetic correlation between EMA and BF in Hanwoo was 0.31 and the genetic correlation between BF and MS was 0.36, which was different from the results in Chikso showing negative genetic correlations. However, Roh et al [20] used Hanwoo young bulls’ data and reported that the genetic correlation between EMA and BF was −0.17 and the correlation between BF and MS was −0.10. Park et al [8] also reported that the genetic correlation between EMA and BF was −0.20 and the correlation between BF and MS was −0.02, which agreed with the trends of the results in this study.

## CONCLUSION

Phenotypic performance of CW, EMA, and BF in Chikso was lower than those of Hanwoo. This is a natural outcome because of two factors: first, there has been a consistent, large scale effort to improve Hanwoo unlike Chikso, and second, Chikso has a smaller population size. The heritabilities of CW, EMA, BF, and MS in Chikso were estimated as 0.50, 0.37, 0.35, and 0.53, respectively, which were higher than those of Hanwoo. Based on the genetic parameters that were estimated in this study, it is expected that the carcass traits will improve when the livestock research institutes at each province conduct small-scale performance tests and the semen is provided to farmers after selecting proven bulls using the state-of-art selection technique such as genomic selection.

## Figures and Tables

**Table 1:** Number of animals by sex, location, birth year, birth season, age at slaughter and coat color

Items	No.
Sex	
Castration	1,134
Male	290
Female	953
Total	2,377
Location	
Gangwon	509
Gyeonggi	160
Gyeongnam	59
Gyeongbuk	264
Jeonbuk	112
Jeonnam	286
Chungnam	257
Chungbuk	730
Total	2,377
Birth year	
2005	27
2006	39
2007	38
2008	65
2009	79
2010	181
2011	397
2012	390
2013	413
2014	448
2015	300
Total	2,377
Birth season	
Spring	926
Summer	685
Fall	399
Winter	367
Total	2,377
Age at slaughter	
1	50
2	1,254
3	401
4	283
50	389
Total	2,377
Coat color	
1	607
2	269
3	353
4	655
5	103
6	296
7	94
Total	2,377

**Table 2:** Distribution of culling Chikso by sex and age

Age	Sex			

	Castration	Male	Female	Sum
0	7 (7.07%)	41 (41.41%)	51 (51.52%)	99
1	15 (25.42%)	11 (18.64%)	33 (55.93%)	59
2	7 (15.22%)	18 (39.13%)	21 (45.65%)	46
3	12 (29.27%)	9 (21.95%)	20 (47.78%	41
4	2 (13.33%)	3 (20.00%)	10 (66.67%)	15
5+	2 (2.63%)	5 (6.58%)	69 (90.79%)	76
Sum	45	87	204	336

(), Row percent.

**Table 3:** Basic statistics of traits

Traits	No.	Mean	SD	Minimum	Maximum
CW	2,377	372.6	63.80	196	557
EMA	2,377	81.97	10.26	53	109
BF	2,377	11.7	5.30	1	25
MS	2,377	3.4	2.01	1	9

SD, standard deviation; CW, carcass weight (kg); EMA, eye muscle area (cm^2^); BF, backfat thickness (mm), MS, marbling score (point).

**Table 4:** Analysis of variances of the carcass traits

Source	DF	CW	EMA	BF	MS
Sex	2	956,799.98**	10,736.11**	5,053.04**	758.03**
Location	7	41,355.85**	311.23**	401.02**	62.33**
Birth year	10	8,243.04**	53.42ns	61.80**	14.35**
Birth season	3	1,676.48ns	238.26*	21.42ns	9.21*
Age at slaughter	4	80,659.01**	832.61**	66.76*	2.81ns
Coat color	6	4,095.61ns	62.67ns	28.97ns	6.74*
Error	2,376	2,406.68	90.51	21.26	2.82

DF, degree of freedom; CW, carcass weight (kg); EMA, eye muscle area (cm^2^); BF, backfat thickness (mm); MS, marbling score (point)

* p<0.05;

** p<0.01; ns, not significant at 0.05 level of significance.

**Table 5:** Least squares means and standard errors of traits by sex

Sex	CW	EMA	BF	MS
Castration	404.85±3.52^a^	83.94±0.68^b^	12.32±0.33^a^	4.36±0.12^a^
Male	404.84±3.73^a^	87.43±0.72^a^	5.04±0.35^b^	1.37±0.13^c^
Female	315.24±3.30^b^	76.14±0.64^c^	11.92±0.31^a^	3.40±0.11^b^

CW, carcass weight (kg); EMA, eye muscle area (cm^2^); BF, backfat thickness (mm); MS, marbling score (point).

a–c Different letters means significant differences among groups (p<0.05).

**Table 6:** Least squares means and standard errors of traits by location

Location	CW	EMA	BF	MS
Gangwon	370.95±3.29^bc^	83.70±0.64^a^	8.46±0.31^b^	3.03±0.11^bc^
Gyeonggi	372.94±4.63^bc^	81.49±0.90^b^	11.67±0.44^a^	3.34±0.16^ab^
Gyeongnam	396.94±7.06^a^	82.98±1.37^ab^	10.15±0.66^ab^	2.73±0.24bcd
Gyeongbuk	361.07±3.96bcd	82.10±0.77^ab^	11.63±0.37^a^	3.73±0.14^a^
Jeonbuk	407.03±5.63^a^	82.39±1.09^ab^	8.38±0.53^b^	3.14±0.19abc
Jeonnam	359.61±3.85^cd^	81.86±0.75^ab^	9.36±0.36^b^	2.66±0.13^cd^
Chungnam	373.02±3.96^b^	83.98±0.77^a^	9.51±0.37^b^	3.33±0.14^ab^
Chungbuk	358.24±3.08^d^	81.54±0.60^b^	8.92±0.29^b^	2.41±0.11^d^

CW, carcass weight (kg); EMA, eye muscle area (cm^2^); BF, backfat thickness (mm); MS, marbling score (point).

a–d Different letters means significant differences among groups (p<0.05).

**Table 7:** Basic statistics of castrated animals by age at slaughter (months)

Age at slaughter	No.	Traits	Mean	SD	Min	Max
28	109	CW	386.5	40.3	281	498
		EMA	83.3	9.0	65	105
		BF	11.8	4.1	4	23
		MS	3.8	1.7	1	9
29	155	CW	391.7	47.3	215	520
		EMA	83.2	9.9	54	108
		BF	12.6	4.5	3	25
		MS	3.7	1.8	1	9
30	175	CW	394.9	45.7	255	501
		EMA	83.6	9.0	62	108
		BF	13.5	4.9	4	25
		MS	4.1	2.0	1	9
31	147	CW	402.0	42.7	296	525
		EMA	83.2	8.9	63	109
		BF	14.0	5.2	3	25
		MS	4.3	1.8	1	9
32	118	CW	400.8	46.0	242	547
		EMA	85.5	8.1	67	105
		BF	12.7	4.7	4	24
		MS	4.7	1.9	1	9

SD, standard deviation; CW, carcass weight (kg); EMA, eye muscle area (cm^2^); BF, backfat thickness (mm); MS, marbling score (point).

**Table 8:** Heritabilities (diagonal), genetic correlations (upper diagonal), and phenotypic correlations (lower diagonal) of carcass traits in Chikso

Traits	CW	EMA	BF	MS
CW	0.50	0.47	0.60	0.09
EMA	0.62	0.37	−0.11	0.36
BF	0.22	0.06	0.35	−0.12
MS	0.18	0.21	0.34	0.53

CW, carcass weight (kg); EMA, eye muscle area (cm^2^); BF, backfat thickness (mm); MS, marbling score (point).
